# Strengths and Weaknesses of Different Therapeutic Strategies for the Treatment of Patients with Multiple Myeloma Who Progress After the Frontline Use of Lenalidomide: A Narrative Review

**DOI:** 10.3390/jcm13206238

**Published:** 2024-10-19

**Authors:** Giuseppe Mele, Nicola Sgherza, Domenico Pastore, Pellegrino Musto

**Affiliations:** 1 Hematology and Stem Cell Transplantation Unit, “Antonio Perrino” Hospital, 72100 Brindisi, Italy; giuseppemele2007@gmail.com (G.M.); domenico.pastore0@gmail.com (D.P.); 2 Hematology and Stem Cell Transplantation Unit, AOUC Policlinico, 70124 Bari, Italy; nicolasgherza@libero.it; 3 Department of Precision and Regenerative Medicine and Ionian Area, “Aldo Moro” University School of Medicine, 70124 Bari, Italy

**Keywords:** relapsed/refractory multiple myeloma, first relapse, second-line treatment, lenalidomide-exposed, lenalidomide-refractory

## Abstract

**Background/Objectives:** Patients with multiple myeloma (MM) who relapse after exposure to lenalidomide in the context of their first-line therapy are becoming a growing and clinically relevant population. We performed a systematic review of available clinical trials evaluating the efficacy and safety of different therapeutic strategies for the treatment of patients with MM at first relapse after the frontline use of lenalidomide. **Methods:** Publications of interest were searched on the PubMed database. The following search terms were employed: relapsed multiple myeloma, refractory multiple myeloma, first relapse, second-line therapy, lenalidomide-refractory (Len-R) and lenalidomide-exposed (Len-Exp). **Results:** Overall, triplet regimens that included anti-CD38 antibodies, carfilzomib and dexamethasone achieved a more favorable PFS regardless of the number of prior therapies. Other trials also demonstrated a non-negligible benefit with combinations containing pomalidomide, particularly in early lines of therapy. However, the variable number of patients with Len-Exp/Len-R disease enrolled in these studies and the limited number of those analyzed after progression following frontline lenalidomide make it difficult to select an “optimal” choice for the treatment of patients with MM at first relapse. Promising results have been more recently obtained by using combo therapies, including belantamab mafodotin and, above all, immunotherapies with CAR-T cells, and ongoing clinical trials are exploring the role of bispecific antibodies and CELMoDs in this population of patients. **Conclusions:** In the absence of clear-cut data regarding the specific effects of available regimens on patients with MM who are refractory or have relapsed after first-line therapies including lenalidomide, novel approaches based on different types of immune strategies are expected to further improve the clinical outcome of these patients.

## 1. Introduction

Lenalidomide, an immunomodulatory drug (IMID) that has CBRL (cereblon) protein as the target for its anti-neoplastic activities, remains a fundamental component of the large majority of multi-agent treatments recommended in the first-line therapy of both patients with transplant-eligible (TE) and non-transplant-eligible (NTE) multiple myeloma (MM) [1]. Therefore, resistance to lenalidomide has become, in recent years, a relevant issue for the choice of appropriate approaches in the context of patients with relapsed/refractory MM (RRMM), particularly at first relapse. Aiming to better clarify this issue, we conducted herein a systematic literature review to provide an updated focus on the optimal positioning and selection of different therapeutic strategies that are now available or will probably be available in the near future in this specific context.

## 2. Material and Methods

Publications of interest were searched on the PubMed database. The following search terms were employed: multiple myeloma, first relapse, second-line therapy, lenalidomide-refractory patients and lenalidomide-exposed patients. Abstracts published by international conferences [American Society of Clinical Oncology (ASCO), American Society of Hematology (ASH), European Hematology Association (EHA) and International Myeloma Society (IMS)], as well as pertinent meta-analyses, were also searched for further relevant studies. The pivotal randomized phase 3 clinical trials identified in the systematic literature review were supplemented with more recent analyses that provided longer follow-up time.

## 3. Results and Discussion

The results of the most important trials leading to the current or plausible next approvals of specific treatments for RRMM and including patients evaluated at first relapse, as well as their possible limitations, are summarized in Table 1 and Table 2.

### 3.1. Monoclonal Antibodies and Proteasome Inhibitor-Based Regimens

In the past decade, the combinations of carfilzomib plus dexamethasone (Kd) (ENDEAVOR phase 3 trial–NCT01568866) [6,7] and daratumumab plus bortezomib and dexamethasone (DaraVd) (CASTOR phase 3 trial–NCT02136134) [2,3,4,5] have both been investigated in comparison with bortezomib and dexamethasone (Vd) in patients with NTE MM previously treated with lenalidomide plus dexamethasone (Rd) upfront, as well as in patients who had received lenalidomide maintenance after autologous stem cell transplantation (ASCT). Specifically, in the ENDEAVOR trial [6,7], 464 patients with RRMM were assigned to receive Kd after a median number of 2 previous lines of therapy (LOTs) (range 1–2). In this group, the number of patients with lenalidomide-refractory (Len-R) disease was 113 (24.0%) regardless of previous LOTs. In the intention-to-treat (ITT) population, the median PFS was 18.7 months, but in patients with Len-R disease, it was only 8.6 months. Regarding the safety profile, among the most relevant grade 3 or 4 treatment-emergent adverse events (TEAEs), anemia and hypertension were found to be slightly more common with Kd than with Vd; conversely, the use of carfilzomib did not result in a higher rate of pneumonia and thrombocytopenia.

In the CASTOR trial [2,3,4,5], 251 patients with more heavily treated RRMM (a median number of 2 previous LOTs, range 1–9) were assigned to receive DaraVd. In this study, the number of patients with Len-R disease was 60 (24.0%) regardless of prior LOTs. After a median follow-up of 47 months, among patients who had received only one previous LOT, the median PFS was 27.0 vs. 7.9 months for DaraVd vs. Vd, respectively (*p* < 0.0001). The PFS benefit for DaraVd vs. Vd was maintained among patients who had received lenalidomide within the first-line therapy (median: 21.2 vs. 7.0 months), but it was significantly lower (7.8 months) in patients with Len-R disease receiving DaraVd. The PFS2 of patients was also significantly prolonged with DaraVd vs. Vd as the first salvage therapy (median: not reached vs. 23.4 months). Notably, the incidence of grade 3–4 upper respiratory tract infections and pneumonia was similar in the two groups, and no increases in toxicities were observed in patients aged ≥75 years in the DaraVd group.

In both these initial studies, however, only a limited fraction of patients with Len-Exp (19.0–38.0%) or Len-R (24.0%) MM who had been pre-treated with lenalidomide were included [2,3,4,5,6,7]. Furthermore, another major limitation was the number of patients who had progressed on frontline lenalidomide that was not specified. Our real-world data on the use of DaraVd in patients with Len-R myeloma obtained by examining patients progressing on lenalidomide following a median number of 2.5 LOTs (range 1–5, with a median time to progression of 11.0 months) are in line with the results presented in the CASTOR trial [29].

More recently, the IKEMA (NCT032275285) [8,9,10,11,12,13] and CANDOR (NCT03158688) (15–17) phase 3 trials included patients with MM with one or two previous LOTs, with higher percentages of patients with Len-R MM (32.0%), although very few were Len-R patients following a single LOT. Going into more detail, in the IKEMA phase 3 trial [8,9,10,11,12,13], 179 patients with RRMM who had received a median number of 2 previous LOTs (range 1–2) were assigned to receive IsaKd vs. Kd. In total, 40% of patients (n. 72) were Len-Exp and 57 patients (32.0%) were Len-R; the number of patients who had progressed on frontline lenalidomide was, indeed, very small (n = 8; 4.4%). After a follow-up of 44 months, in the ITT population, the median PFS was 35.7 months. The median PFS for patients with Len-R MM progressing on frontline lenalidomide was not evaluated due to the very small number of patients, but the HR for the PFS benefit in patients with Len-R MM, regardless of the number of previous LOTs, was similar to the HR in the ITT population (0.59 vs. 0.58, respectively). Notably, in a recent update to this trial, no difference in overall survival could be detected between the treatment groups, despite a significant improvement with IsaKd in terms of time-to-next-treatment and second-PFS being observed. Regarding TEAEs, IsaKd was associated with a higher percentage of grade ≥3 neutropenia, upper respiratory tract infections, pneumonia and bronchitis, particularly in patients aged >70 years. The authors concluded that IsaKd might be considered an important treatment option for patients with myeloma with Len-R disease. However, the limitations of this study regarding the topic of the present review were (i) the small number of patients with Len-R disease enrolled; (ii) the very limited number of patients progressing on frontline lenalidomide; and (iii) the fact that the PFS of the patients with Len-R disease was unknown. We are currently exploring the role of IsaKD in patients with MM with Len-R or Len-Exp disease at first relapse in a real-life, multicenter study (paper in preparation).

In the CANDOR phase 3 trial [14,15,16], DaraKd was administered in 312 patients with RRMM who had received 1–3 prior LOTs and, again, compared with Kd; 123 patients (39.0%) were Len-Exp and 99 patients (32.0%) were Len-R to any previous lenalidomide-including regimen. The median PFS was 28.4 months in the entire DaraKd group, whereas it was not reached for patients who were Len-Exp and was 28 months for patients who were Len-R. DaraKd resulted in a PFS benefit both among patients with Len-Exp and those with Len-R disease regardless of the number of previous LOTs. All grades of upper respiratory tract infections, thrombocytopenia and anemia occurred with a moderately higher incidence in the DaraKd group than in the Kd group, as well as grade ≥3 pneumonia. However, the frequency of TEAEs leading to treatment discontinuation was similar in both groups, and five deaths (mainly due to infections) were reported as treatment-related, all in the DaraKd group. Again, the exact number of patients progressing on frontline lenalidomide was unspecified in this study.

### 3.2. Monoclonal Antibodies and Pomalidomide Based-Regimens

The OPTIMISMM (NCT01734928) [17] and APOLLO (NCT03180736) [18] phase 3 trials were specifically designed to include the growing and clinically relevant population of patients with Len-R disease (from 71.0% to 80.0%). Overall, these studies demonstrated that the combination of pomalidomide with bortezomib and dexamethasone (PVd), daratumumab with pomalidomide and dexamethasone (DaraPd) may represent effective options for patients with RRMM.

In the OPTIMISMM phase 3 trial [17], 281 patients with Len-Exp RRMM who had received a median number of 2 LOTs (range 1–3) were assigned to receive PVd and compared with Vd, 40% of whom had received more than 1 prior LOT. With a median follow-up of 16 months, the median PFS was 11.2 months in the ITT population. The median PFS for the subset of patients with Len-R disease was 9.5 months. Notably, the median PFS of the patients with Len-R disease who had progressed after frontline lenalidomide-based therapy was 17.8 months. The most common grade 3 or 4 TEAEs were neutropenia and infections, both significantly more frequent in patients who received PVd.

In the APOLLO phase 3 trial [18], DaraPd was tested and compared with pomalidomide plus dexamethasone (Pd) in 151 patients with Len-Exp disease, 120 of whom (79.0%) had Len-R disease; only 16 of these patients (11.0%), however, had received a single previous treatment. Indeed, the large majority of patients (89.0%; n = 135) were more heavily pre-treated and had undergone a median of 2 previous LOTs (range 1–5). After a median follow-up of 16.9 months, in the ITT population, the median PFS was 12.4 months, whereas the median PFS for the patients with Len-R disease was 9.9 months regardless of the number of LOTs previously received. The most frequent grade 3–4 TEAEs were neutropenia and infections, which were slightly more represented in the DaraPd group, where pneumonia was also the most common serious TEAE. The important limitations of the study were (i) the significant number of prior treatments; (ii) the very small number of patients with Len-R disease who had progressed on frontline lenalidomide; and (iii) the fact that the median PFS of the patients with Len-R disease who had progressed on frontline lenalidomide was unknown. Interestingly, Weisel et al. [19] recently performed a MAIC (matching-adjusted indirect comparison) analysis of the lenalidomide-sparing CANDOR vs. OPTIMISMM and APOLLO phase 3 trials. They demonstrated that DaraKd performed significantly better than PVd and DaraPd in terms of PFS (PFS: 22.6 months DaraKd vs. 11.2 months PVd; PFS: 21.7 months DaraKd vs. 12.1 months DaraPd) in patients with Len-Exp RRMM, the majority of whom were Len-R. Unfortunately, in this indirect comparison, sample sizes were insufficient to perform robust analyses in patients who had received only one prior line of therapy.

The same DaraPd combination was investigated in the MM-014 phase 2 trial (NCT01946477) [30]. This study included 112 patients who were Len-Exp, 84 of whom (75.0%) had Len-R disease and 70 of whom (62.5%) had received only one previous therapy. This phase 2 trial was, therefore, predominantly designed for patients with Len-R disease in relapse after frontline therapy [ASCT followed by consolidation and lenalidomide maintenance until progression or DaraRd/VRd (daratumumab + lenalidomide + dexamethasone/bortezomib + lenalidomide + dexamethasone) for patients with NTE newly diagnosed with MM]. Regarding ITT, the median PFS was 30.8 months, whereas the median PFS in patients with Len-R disease was 23.7 months independently of the number of prior LOTs. Infections and neutropenia of any grade were the most common TEAEs, with grade 3–4 infections (mainly pneumonia) occurring in more than one-third of patients. Although the median PFS of patients progressing on frontline lenalidomide was not specified, the promising results presented in this non-randomized trial are in line with real-world data obtained by examining patients progressing on lenalidomide following one prior line of treatment, where DaraPd was associated with a median PFS of 18.9 months in a population that had relapsed on lenalidomide maintenance post ASCT [31].

Phase 1/2 studies [20,32,33] have demonstrated the high efficacy of combinations with pomalidomide plus carfilzomib and dexamethasone (KPd) in over 200 patients with Len-R disease. In particular, the EMN11 phase 2 trial [20] (EudraCT 2013-003265-34) investigated reinduction with KPd, followed by continuous pomalidomide +/− dexamethasone in 112 patients with Len-R MM at first progression after frontline therapy including or not including ASCT, but all receiving lenalidomide maintenance. At a follow-up of 40 months, the median PFS was 32 months for patients who received KPd plus ASCT (n = 35) and 17 months for those without ASCT (n = 76), who, however, could receive ASCT as salvage treatment. The PFS was better after a longer duration of prior lenalidomide, and the median OS was 67 months. The KPd-emerging grade 3/4 AEs mainly included cytopenia, infections and cardiovascular toxicities.

### 3.3. Other Treatments and Future Perspectives

Regarding novel drugs, selinexor in combination with bortezomib and dexamethasone (SVd) was compared to Vd in 195 patients with RRMM who had received from one to three prior therapies in the BOSTON phase 3 trial (NCT03110562) [21]; 77 patients (39.0%) were Len-Exp and 53 (27.0%) were Len-R. In a subgroup analysis of the study, after a median follow-up of approximately 29 months, Mateos et al. showed that the median PFS was 10.2 months in the SVd group for patients who were Len-R in any previous LOTs [22]; but, again, the number of patients who had progressed after frontline lenalidomide was unknown. Regarding grade 3/4 AEs, thrombocytopenia, anemia and fatigue were more frequent with SVd, as well as grade 1/2 diarrhea, whereas the incidence of pneumonia and the number of deaths were similar in the two groups.

The pivotal, randomized, phase 3 trials DREAMM-7 (NCT04246047) [23,24] [belantamab, bortezomib and desamethasone (BVd) vs. DaraVd] and DREAMM-8 (NCT04484623) [25] [belantamab, pomalidomide and dexamethasone (BPd) vs. PVd] investigated combinations containing the drug-conjugated, anti-BCMA monoclonal antibody belantamab mafodotin and were mainly designed for patients with Len-Exp and Len-R RRMM after one to more than four previous LOTs.

In the DREAMM-7 study [23,24], 127 patients with RRMM (52%) enrolled in the BVd arm had previously received lenalidomide (Len-Exp), and 79 (33.0%) were Len-R. Twenty-two patients had undergone a single treatment before enrolment in the study. After a median follow-up of 28.2 months, the study demonstrated the superiority of BVd regardless of the number of previous LOTs (PFS 36.6 months in the ITT population and 25.0 months in patients with Len-R disease). Among the AEs, the incidence of any grade of thrombocytopenia and ocular events, as well as that of grade ≥3 pneumonia, was significantly higher in the BVd group than in the DVd group. In a post hoc analysis, immunoglobulin replacement was also more common with BVd.

In the DREAMM-8 study [25], 155 patients were assigned to the BPd group; all of them were Len-Exp, and 81.0% were Len-R. At a median follow-up of 21.8 months, the 12-month estimated PFS with BPd was 71.0%, significantly better than for PVd. In a recent update to the study [26], Trudel et al. showed that the median PFS was not reached in the ITT population treated with BPd, whereas the median PFS for the subset of patients with Len-R disease was 24 months, regardless of the number of LOTs, thus demonstrating that the PFS benefit observed in the Len-R subgroup was consistent with that seen in the overall study population. The most frequent AEs in the BPd group were blurred vision, dry eyes and a foreign-body sensation in the eyes. Infection of grade ≥3 also occurred more frequently in the BPd group. Unfortunately, in terms of the effects at first relapse after lenalidomide therapy, these studies also have limitations: in the DREAMM-7 trial, the PFS of patients who had progressed on frontline lenalidomide was unknown, whereas in the DREAMM-8 study, the percentage of patients with Len-R disease following only one LOT and the PFS of patients with Len-R disease were not reported.

T-cell-redirecting immune therapies with bispecific antibodies recognizing BCMA (i.e., teclistamab), GPRC5D (i.e., talquetamab) or other targets (i.e., FcRH5, cevostamab) are emerging as highly effective treatments for patients with RRMM, particularly in those with Len-R disease. Notably, the ongoing trials MajesTEC-9 [teclistamab vs. elotuzumab, pomalidomide and dexamethasone (EloPd) or Kd] (NCT05572515) and MONUMENTAL-6 (talquetamab plus teclistamab or talquetamab plus pomalidomide vs. EloPd/PVd) (NCT06208150) include patients with Len-Exp and Len-R RRMM after one or more LOTs. The results from these studies are not yet published, but those relating to patients at first relapse with Len-Exp and Len-R MM could be of particular interest.

Regarding the use of CAR-T cells, the randomized CARTITUDE-4 phase 3 trial (cilta-cel vs. standard of care) demonstrated the impressive superiority of anti-BCMA CAR-T cells in patients with Len-R disease who had received from one to three prior LOTs [27], including DaraPd and PVd [28]. At 12 months, in fact, the median PFS was not reached in the cilta-cel group, either in patients in first progression after frontline lenalidomide-based therapy or in those who had received more than one previous LOT. A primary interim analysis confirmed that the grade 3/4 neutropenia did not differ substantially between CAR-T therapy and the other standard treatments employed, whereas the incidence of similar grade thrombocytopenia and anemia was higher in the cilta-cel group than in the control group. Notably, grade 3 or higher infections were similar in the two groups. CAR-T therapy based on novel constructs and/or directed against additional or multiple targets is currently being explored in patients with RRMM.

Finally, the activity of CELMoDs iberdomide and mezigdomide (selected S-isomer, more selective anti-cereblon molecules), as single agents or in combination with other drugs, is also under clinical investigation in RRMM [34]; however, at present, no data are available in the specific context of patients at first relapse [35].

## 4. Conclusions

In this narrative review, we tried to evaluate the strengths and weaknesses of the published therapeutic strategies for the treatment of patients with MM who progress after the frontline use of lenalidomide. Possible criticisms of this type of study design and uncertainties in the analysis of statistics and trial concepts are probably appropriate. However, it is a fact that no phase 3 pivotal studies have been so far exclusively conducted in patients with Len-Exp/Len-R RRMM following only one LOT. A possible increase in grade 3–4 AEs with novel treatments (i.e., infections and, particularly, pneumonia) or drug-specific toxicities (i.e., ocular toxicity in belantamab-treated patients), although generally acceptable and manageable, should also be taken in consideration. All this makes it difficult to select an “optimal” choice for the treatment of these patients at first relapse, despite triplet regimens, including anti-CD38 antibodies, carfilzomib and dexamethasone, having achieved more significant PFS (however, regardless of the number of prior therapies). This observation would support the idea that a switch in the class of agent could be preferable following lenalidomide failure, although the OPTIMISMM [17], MM014 [19] and EMN11 [20] trials also demonstrated a non-negligible benefit with combinations containing pomalidomide, particularly in early lines of therapy in RRMM. Indeed, the available data should be interpreted with caution due to differences in the trial designs, the schedules and doses of lenalidomide employed, and the numerical differences in the patient population selection, particularly regarding the variable number (including an appropriate definition) of patients with Len-Exp/Len-R disease enrolled and the limited number of those analyzed after progression following frontline lenalidomide. In particular, one of the most relevant issues resides in the different starting dose of lenalidomide. In the MM-014 trial, for example, the most common lenalidomide dose was ≤10 mg (48.2% of patients) [19], whereas in the APOLLO trial, the most common dose administered varied from 15 to 25 mg (71.7% of patients) [18]. In other studies, the exact dosage of lenalidomide was not even specified. Based on recent data, the positive effects achieved with combo therapies, including belantamab mafodotin, could be of particular interest for the growing population of patients relapsed after both daratumumab and lenalidomide employed as frontline therapy. On the other hand, immunotherapies with CAR-T cells would seem to be the most promising approach for these patients. The role of bispecific antibodies and that of CELMoD agents warrant evaluation in patients with MM at first relapse after the initial use of lenalidomide, and the results of ongoing studies could provide evidence about the efficacy of these further approaches in this specific population of patients.

## Figures and Tables

**Table 1:** Main prior clinical trials including patients with lenalidomide-exposed/refractory myeloma at first relapse.

	**Proteasome Inhibitor-Containing**			
	**CASTOR** [2,3,4,5]	**ENDEAVOR** [6,7]	**IKEMA** [8,9,10,11,12,13]	**CANDOR** [14,15,16]
**Drugs**	DaraVd	Kd	IsaKd	DaraKd
**Len-Exp %** **(n. of patients)**	36 (90)	38 (177)	40 (72)	39 (123)
**Len-R %** **(n. of patients)**	24 (60)	24 (113)	32 (57) 1 prior LOT 14 (8)	32 (99)
**Median n. of previous lines of treatment (range)**	2 (1–9)	2 (1–2)	2 (1–2)	2 (1–3)
**ORR**	82.9%	77%	87%	84%
**mPFS ITT**	16.7 months	18.7 months	NR	35.7 months HR 0.58
**mPFS Len-R** **regardless of the n. of prior therapies**	7.8 months	8.6 months	HR 0.59	NR
**mPFS Len-R** **after 1 previous line of therapy**	/	/	/	/
**Limitations**	heavily pre-treated patients	n. of patients progressing on frontline lenalidomide unspecified	heavily pre-treated patients	heavily pre-treated patients
	n. of patients progressing on frontline lenalidomide unspecified		very small n. of patients progressing on frontline lenalidomide (n = 8)	n. of patients progressing on frontline lenalidomide unspecified
			PFS of patients with Len-R disease unspecified	
	**Pomalidomide-Containing**			
	**OPTIMISMM** [17]	**APOLLO** [18]	**MM014** [19]	**EMN011** [20]
**Drugs**	PVd	DaraPd	DaraPd	KPd
**Len-Exp %** **(n. of patients)**	100 (281)	100 (151)	100 (112)	100 (112)
**Len-R %** **(n. of patients)**	71 (200) 1 prior LOT 58 (64)	80 (120) 1 prior LOT 11 (16)	75 (84) 1 prior LOT 62.5 (70)	100 (112)
**Median number of previous LOTs (range)**	2 (1–3)	2 (1–5)	1 (1–2)	1
**Median PFS ITT**	11.2 months	12.4 months	30.8 months	/
**Median PFS Len-R** **regardless of the number of previous LOTs**	9.5 months	9.9 months	23.7 months	/
**Median PFS Len-R** **after 1 previous LOT**	17.8 months	/	/	26 months (with ASCT: 32.0 months) (without ASCT: 17.0 months)
**Limitations**	heavily pre-treated patients	heavily pre-treated patients	design study (phase 2 trial)	design study (phase 2 trial)
		small number of patients with Len-R disease progressing on frontline lenalidomide (n = 16)	PFS of patients with Len-R disease progressing on frontline lenalidomide unspecified	
		PFS of patients with Len-R disease progressing on frontline lenalidomide unspecified		

Legend: ASCT: autologous stem cell transplantation; DaraKd: daratumumab, carfilzomib, dexamethasone; DaraPd: daratumumab, pomalidomide, dexamethasone; DaraVd: daratumumab, bortezomib, dexamethasone; HR: hazard ratio; IsaKd: isatuximab, carfilzomib, dexamethasone; ITT: intention-to-treatment; KPd: carfilzomib, pomalidomide, dexamethasone; Len-Exp: lenalidomide exposed; Len-R: lenalidomide-refractory; LOT: line of therapy; NR: not reached; PFS: progression-free survival; PVd: pomalidomide, bortezomib, dexamethasone.

**Table 2:** Main clinical trials with novel agents including patients with lenalidomide-exposed/refractory myeloma at first relapse.

	Selinexor-Containing	Belantamab-Containing		Cilta Cell-Containing
	BOSTON [21,22]	DREAMM-7 [23,24]	DREAMM-8 [25,26]	CARTITUDE-4 [27,28]
**Drugs**	SVd	BVd	BPd	Cilta-cel
**Len-Exp (%)** **(n. of patients)**	39 (77)	52 (127)	100 (155)	100 (208)
**Len-R (%)** **(n. of patients)**	27 (53)	33 (79) 1 prior LOT 28 (22)	81 (125)	100 (208) (inclusion criteria: refractory to lenalidomide)
**Median number of previous LOTs (range)**	2 (1–3)	From 1 to ≥4	From 1 to ≥4	From 1 to 3
**Media PFS ITT**	13.93 months	36.6 months	NR	NR
**Median PFS Len-R** **regardless of the number of previous LOTs**	10.2 months	25 months	/	NR
**Median PFS Len-R** **after 1 previous LOT**	/	/	/	NR
**Limitations**	Heavily pre-treated patients	Heavily pre-treated patients	Heavily pre-treated patients	/
	PFS of patients with Len-R disease progressing on frontline lenalidomide unspecified	PFS of patients with Len-R disease unspecified	PFS of patients with Len-R disease unspecified	

Legend: BPd: belantamab, pomalidomide, dexamethasone; BVd: belantamab, bortezomib, dexamethasone; ITT: intention-to-treatment; Len-Exp: lenalidomide exposed; Len-R: lenalidomide-refractory; LOT: line of therapy; PFS: progression-free survival; NR: not reached; SVd: selinexor, bortezomib, dexamethasone.

## Data Availability

Data and materials referenced in this review were derived from previously published studies in the literature. All included studies are listed in the References section.

## References

[1] Dimopoulos M.A., Moreau P., Terpos E., Mateos M.V., Zweegman S., Cook G., Delforge M., Hájek R., Schjesvold F., Cavo M. (2021). Multiple Myeloma: EHA-ESMO Clinical Practice Guidelines for Diagnosis, Treatment and Follow-up. Hemasphere.

[2] Palumbo A., Chanan-Khan A., Weisel K., Nooka A.K., Masszi T., Beksac M., Spicka I., Hungria V., Munder M., Mateos M.V. (2016). Daratumumab, Bortezomib, and Dexamethasone for Multiple Myeloma. N. Engl. J. Med..

[3] Usmani S.Z., Mateos M.V., Lentzsch S., Quach H., Capra M., Ovilla R., Jo J.C., Shin H.J., Sonneveld P., Qi M. (2018). Efficacy of daratumumab in combination with standard of care regimens in lenalidomide-exposed or refractory patients with relapsed/refractory multiple myeloma (RRMM): Analysis of the CASTOR, POLLUX and MMY1001 studies. Blood.

[4] Mateos M.V., Sonneveld P., Hungria V., Nooka A.K., Estell J.A., Barreto W., Corradini P., Min C.K., Medvedova E., Weisel K. (2020). Daratumumab, bortezomib, and dexamethasone versus bortezomib and dexamethasone in patients with previously treated multiple myeloma: Three-year follow-up of CASTOR. Clin. Lymphoma Myeloma Leukemia.

[5] Weisel K.C., Sonneveld P., Mateos M.V., Hungria V.T.M., Spencer A., Estell J., Barreto W.G., Corradini P., Min C.K., Medvedova E. (2019). Efficacy and Safety of Daratumumab, Bortezomib, and Dexamethasone (D-Vd) Versus Bortezomib and Dexamethasone (Vd) in First Relapse Patients (pts) with Multiple Myeloma (MM): Four-Year Update of Castor. Blood.

[6] Dimopoulos M.A., Moreau P., Palumbo A., Joshua D., Pour L., Hájek R., Facon T., Ludwig H., Oriol A., Goldschmidt H. (2016). Carfilzomib and dexamethasone versus bortezomib and dexamethasone for patients with relapsed or refractory multiple myeloma (ENDEAVOR): A randomised, phase 3, open-label, multicentre study. Lancet Oncol..

[7] Moreau P., Joshua D., Chng W.J., Palumbo A., Goldschmidt H., Hájek R., Facon T., Ludwig H., Pour L., Niesvizky R. (2017). Impact of prior treatment on patients with relapsed multiple myeloma treated with carfilzomib and dexamethasone vs bortezomib and dexamethasone in the phase 3 ENDEAVOR study. Leukemia.

[8] Moreau P., Dimopoulos M.A., Mikhael J., Yong K., Capra M., Facon T., Hajek R., Špička I., Baker R., Kim K. (2021). Isatuximab, carfilzomib, and dexamethasone in relapsed multiple myeloma (IKEMA): A multicentre, open-label, randomised phase 3 trial. Lancet.

[9] Moreau P., Dimopoulos M.A., Mikhael J., Yong K., Capra M., Facon T., Hajek R., Špička I., Casca F., Mace S. (2022). Updated progression-free survival and depth of response in IKEMA, a randomized phase 3 trial of isatuximab, carfilzomib, and dexamethasone (Isa-Kd) vs Kd in relapsed multiple myeloma. Ann Oncol..

[10] Dimopoulos M.A., Moreau P., Augustson B., Castro N., Pika T., Delimpasi S., De la Rubia J., Maiolino A., Reiman T., Martinez-Lopez J. (2023). Isatuximab plus carfilzomib and dexamethasone in patients with relapsed multiple myeloma based on prior lines of treatment and refractory status: IKEMA subgroup analysis. Am. J. Hematol..

[11] Martin T., Dimopoulos M.A., Mikhael J., Yong K., Capra M., Facon T., Hajek R., Špička I., Baker R., Kim K. (2023). Isatuximab, carfilzomib, and dexamethasone in patients with relapsed multiple myeloma: Updated results from IKEMA, a randomized Phase 3 study. Blood Cancer J..

[12] Facon T., Moreau P., Martin T.G., Špička I., Oriol A., Koh Y., Lim A., Mikala G., Rosiñol L., Yağci M. (2021). Isatuximab plus carfilzomib and dexamethasone versus carfilzomib and dexamethasone in elderly patients with relapsed multiple myeloma: IKEMA subgroup analysis. J. Clin. Oncol..

[13] Yong K., Martin T., Dimopoulos M.A., Mikhael J., Capra M., Facon T., Hajek R., Špička I., Baker R., Kim K. (2024). Isatuximab plus carfilzomib-dexamethasone versus carfilzomib-dexamethasone in patients with relapsed multiple myeloma (IKEMA): Overall survival analysis of a phase 3, randomised, controlled trial. Lancet Haematol..

[14] Dimopoulos M., Quach H., Mateos M.V., Landgren O., Leleu X., Siegel D., Weisel K., Yang H., Klippel Z., Zahlten-Kumeli A. (2020). Carfilzomib, dexamethasone, and daratumumab versus carfilzomib and dexamethasone for patients with relapsed or refractory multiple myeloma (CANDOR): Results from a randomised, multicentre, open-label, phase 3 study. Lancet.

[15] Usmani S.Z., Quach H., Mateos M.V., Landgren O., Leleu X., Siegel D., Weisel K., Gavriatopoulou M., Oriol A., Rabin N. (2022). Carfilzomib, dexamethasone, and daratumumab versus carfilzomib and dexamethasone for patients with relapsed or refractory multiple myeloma (CANDOR): Updated outcomes from a randomised, multicentre, open-label, phase 3 study. Lancet Oncol..

[16] Usmani S.Z., Quach H., Mateos M.V., Landgren O., Leleu X., Siegel D., Weisel K., Shu X., Li C., Dimopoulos M. (2023). Final analysis of carfilzomib, dexamethasone, and daratumumab vs carfilzomib and dexamethasone in the CANDOR study. Blood Adv..

[17] Richardson P.G., Oriol A., Beksac M., Liberati A.M., Galli M., Schjesvold F., Lindsay J., Weisel K., White D., Facon T. (2019). Pomalidomide, bortezomib, and dexamethasone for patients with relapsed or refractory multiple myeloma previously treated with lenalidomide (OPTIMISMM): A randomised, open-label, phase 3 trial. Lancet Oncol..

[18] Dimopoulos M.A., Terpos E., Boccadoro M., Delimpasi S., Beksac M., Katodritou E., Moreau P., Baldini L., Symeonidis A., Bila J. (2021). Daratumumab plus pomalidomide and dexamethasone versus pomalidomide and dexamethasone alone in previously treated multiple myeloma (APOLLO): An open-label, randomised, phase 3 trial. Lancet Oncol..

[19] Weisel K., Dimopoulos M.A., Beksac M., Leleu X., Richter J., Heeg B., Patel S., Majer I., McFadden I., Mikhael J. (2024). Carfilzomib, daratumumab, and dexamethasone (KdD) vs. lenalidomide-sparing pomalidomide-containing triplet regimens for relapsed/refractory multiple myeloma: An indirect treatment comparison. Leuk. Lymphoma.

[20] Sonneveld P., Zweegman S., Cavo M., Nasserinejad K., Broijl A., Troia R., Pour L., Croockewit S., Corradini P., Patriarca F. (2022). Carfilzomib, Pomalidomide, and Dexamethasone as Second-line Therapy for Lenalidomide refractory Multiple Myeloma. Hemasphere.

[21] Grosicki S., Simonova M., Spicka I., Pour L., Kriachok I., Gavriatopoulou M., Pylypenko H., Auner H.W., Leleu X., Doronin V. (2020). Once-per-week selinexor, bortezomib, and dexamethasone versus twice-per-week bortezomib and dexamethasone in patients with multiple myeloma (BOSTON): A randomised, open-label, phase 3 trial. Lancet.

[22] Mateos M.V., Engelhardt M., Leleu X., Mesa M.G., Auner H.W., Cavo M., Dimopoulos M.A., Bianco M., Merlo G.M., la Porte C. (2023). Efficacy, survival and safety of Selinexor, Bortezomib and Dexamethasone (SVd) in patients witn Lenalidomide-refractory Multiple Myeloma: Subgroup data from the Boston trial. HemaSphere.

[23] Hungria V., Robak P., Hus M., Zherebtsova V., Ward C., Ho P.J., Ribas de Almeida A.C., Hajek R., Kim K., Grosicki S. (2024). DREAMM-7 Investigators. Belantamab Mafodotin, Bortezomib, and Dexamethasone for Multiple Myeloma. N. Engl. J. Med..

[24] Mateos M.V., Robak P., Hus M., Fu C., Zherebtsova V., Ward C., Ho P.J., de Almeida A.C., Hajek R., Kim K. (2024). DREAMM-7 update: Subgroup analyses from a phase 3 trial of belantamab mafodotin + bortezomib and dexamethasone vs daratumumab, bortezomib, and dexamethasone in relapsed/refractory multiple myeloma. J. Clin. Oncol..

[25] Dimopoulos M.A., Beksac M., Pour L., Delimpasi S., Vorobyev V., Quach H., Špička I., Radocha J., Robak P., Kim K. (2024). DREAMM-8 Investigators. Belantamab Mafodotin, Pomalidomide, and Dexamethasone in Multiple Myeloma. N. Engl. J. Med..

[26] Trudel S., Beksac M., Poor L., Delimpasi S., Vorobyev V., Quach H., Sipcka I., Radocha J., Robak P., Kim K. (2024). Result from the randomized phase III DREAMM-8 study of belantamab mafodotin plus pomalidomide and dexamethasone versus pomalidomide plus bortezomib and dexamethasone in relapsed/refractory multiple myeloma. Clin. Lymphoma Myeloma Leuk..

[27] San-Miguel J., Dhakal B., Yong K., Spencer A., Anguille S., Mateos M.V., Fernández de Larrea C., Martínez-López J., Moreau P., Touzeau C. (2023). Cilta-cel or Standard Care in Lenalidomide-Refractory Multiple Myeloma. N. Engl. J. Med..

[28] Einsele H., Yong K., Harrison S., Mateos M.V., Moreau P., van de Donk N.W.C.J., Sidana S., Popat R., Lendvai N., Lonardi C. (2023). Phase 3 results from CARTITUDE-4: Cilta-cel versus standard of care (PVd or DPD) in lenalidomide-refractory multiple myeloma. HemaSphere.

[29] Mele G., Di Renzo N., Cascavilla N., Carella A.M., Guarini A., Mazza P., Melillo L., Pavone V., Tarantini G., Curci P. (2023). Real-world evidence on the use of daratumumab, bortezomib and dexamethasone (DVd) in lenalidomide-refractory myeloma patients: Subgroup analysis of the multicenter retrospective experience by “rete ematologica pugliese”. Leuk. Lymphoma.

[30] Bahlis N.J., Siegel D.S., Schiller G.J., Samaras C., Sebag M., Berdeja J., Ganguly S., Matous J., Song K., Seet C.S. (2022). Pomalidomide, dexamethasone, and daratumumab immediately after lenalidomide-based treatment in patients with multiple myeloma: Updated efficacy, safety, and health-related quality of life results from the phase 2 MM-014 trial. Leuk. Lymphoma.

[31] Mian H., Eisfeld C., Venner C.P., Masih-Khan E., Kardjadj M., Jimenez-Zepeda V.H., Khandanpour C., Lenz G., McCurdy A., Sebag M. (2022). Efficacy of daratumumab-containing regimens among patients with multiple myeloma progressing on lenalidomide maintenance: Retrospective analysis. Front Oncol..

[32] Shah J.J., Stadtmauer E.A., Abonour R., Cohen A.D., Bensinger W.I., Gasparetto C., Kaufman J.L., Lentzsch S., Vogl D.T., Gomes C.L. (2015). Carfilzomib, pomalidomide, and dexamethasone for relapsed or refractory myeloma. Blood.

[33] Bringhen S., Mina R., Cafro A.M., Liberati A.M., Spada S., Belotti A., Gaidano G., Patriarca F., Troia R., Fanin R. (2018). Once-weekly carfilzomib, pomalidomide, and low-dose dexamethasone for relapsed/refractory myeloma: A phase I/II study. Leukemia.

[34] Liu Y., Mo C.C., Hartley-Brown M.A., Sperling A.S., Midha S., Yee A.J., Bianchi G., Piper C., Tattersall A., Nadeem O. (2024). Targeting Ikaros and Aiolos: Reviewing novel protein degraders for the treatment of multiple myeloma, with a focus on iberdomide and mezigdomide. Expert Rev. Hematol..

[35] Richardson P.G., Trudel S., Popat R., Mateos M.V., Vangsted A.J., Ramasamy K., Martinez-Lopez J., Quach H., Orlowski R.Z., Arnao M. (2023). Mezigdomide plus Dexamethasone in Relapsed and Refractory Multiple Myeloma. N. Engl. J. Med..

